# P16 immunohistochemistry is a sensitive and specific surrogate marker for *CDKN2A* homozygous deletion in gliomas

**DOI:** 10.1186/s40478-023-01573-2

**Published:** 2023-05-03

**Authors:** Meenakshi Vij, Benjamin B. Cho, Raquel T. Yokoda, Omid Rashidipour, Melissa Umphlett, Timothy E. Richardson, Nadejda M. Tsankova

**Affiliations:** 1 Department of Pathology, Molecular, and Cell-Based Medicine, Icahn School of Medicine at Mount Sinai, New York, NY 10029 USA; 2 Department of Pathology and Laboratory Medicine, University of North Carolina at Chapel Hill, Chapel Hill, NC USA; 3 Department of Neuroscience, Icahn School of Medicine at Mount Sinai, New York, NY 10029 USA

**Keywords:** CDKN2A homozygous deletion, p16, Immunohistochemistry, Glioma

## Abstract

Molecular characterization of gliomas has uncovered genomic signatures with significant impact on tumor diagnosis and prognostication. *CDKN2A* is a tumor suppressor gene involved in cell cycle control. Homozygous deletion of the *CDKN2A/B* locus has been implicated in both gliomagenesis and tumor progression through dysregulated cell proliferation. In histologically lower grade gliomas, *CDKN2A* homozygous deletion is associated with more aggressive clinical course and is a molecular marker of grade 4 status in the 2021 WHO diagnostic system. Despite its prognostic utility, molecular analysis for *CDKN2A* deletion remains time consuming, expensive, and is not widely available. This study assessed whether semi-quantitative immunohistochemistry for expression of p16, the protein product of *CDKN2A*, can serve as a sensitive and a specific marker for *CDKN2A* homozygous deletion in gliomas. P16 expression was quantified by immunohistochemistry in 100 gliomas, representing both IDH-wildtype and IDH-mutant tumors of all grades, using two independent pathologists’ scores and QuPath digital pathology analysis. Molecular *CDKN2A* status was determined using next-generation DNA sequencing, with homozygous *CDKN2A* deletion detected in 48% of the tumor cohort. Classifying *CDKN2A* status based on p16 tumor cell expression (0–100%) demonstrated robust performance over a wide range of thresholds, with receiver operating characteristic curve area of 0.993 and 0.997 (blinded and unblinded pathologist p16 scores, respectively) and 0.969 (QuPath p16 score). Importantly, in tumors with pathologist-scored p16 equal to or less than 5%, the specificity for predicting *CDKN2A* homozygous deletion was 100%; and in tumors with p16 greater than 20%, specificity for excluding *CDKN2A* homozygous deletion was also 100%. Conversely, tumors with p16 scores of 6–20% represented gray zone with imperfect correlation to *CDKN2A* status. The findings indicate that p16 immunohistochemistry is a reliable surrogate marker of *CDKN2A* homozygous deletion in gliomas, with recommended p16 cutoff scores of ≤ 5% for confirming and > 20% for excluding biallelic *CDKN2A* loss.

**Supplementary Information:**

The online version contains supplementary material available at 10.1186/s40478-023-01573-2.

## Introduction

Until recently, grading of solid tumors was based solely on histology, immunohistochemistry (IHC) and ultrastructural findings. In the past decade, high throughput sequencing technology has enabled the discovery of common and unique single nucleotide variants, large gains and losses, and fusions across many tumors, some of which have impacted tumor categorization and prognostication. Genomic and epigenomic molecular analyses have provided new insights into the mechanisms for tumorigenesis, have enabled the stronger correlation of histology with tumor grade, and have helped distinguish morphologically similar tumors with unique molecular signatures. This is especially true for tumors of the central nervous system (CNS), with the latest (5th) edition of the WHO CNS tumor classification incorporating key molecular alterations into the classification and grading of glial and glioneuronal neoplasms [1].

A notable example of a genomic alteration with evolving prognostic value in brain tumors is the loss of the tumor suppressor gene cyclin dependent kinase inhibitor 2A, *CDKN2A*. Alternative splicing of the *CDKN2A* locus on chromosome 9p21 results in the translation of two main tumor suppressor proteins: the cyclin dependent kinase inhibitor p16 (aka p16INK4A, p16INK4, CDK4I, MTS1) and, through an alternate open reading frame (ARF), the structurally distinct protein p14 (aka p14ARF) [2, 3]. The p16INK4A protein, referred to as p16 hereafter, inhibits abnormal cell growth and proliferation by binding to complexes of cyclin-dependent kinases (CDK) 4 and 6 and cyclin D, thus inhibiting retinoblastoma protein phosphorylation and causing cell cycle arrest in the G1 phase [2]. In contrast, p14 functions to stabilize the tumor suppressor protein p53 and to sequester MDM2, a protein responsible for the degradation of p53. Together, both CDKN2A tumor suppressor proteins help regulate entry into the S phase of the cell cycle. CDKN2A inactivation provides a survival advantage to cancer cells, with the most common genomic alteration causing this event being the homozygous (biallelic) deletion of *CDKN2A*. In greater than 90% of cancer tissues harboring *CDKN2A* deletion, the adjacent *CDKN2B* gene on chromosome 9p, encoding the p15INK4B cyclin dependent kinase inhibitor, is also deleted [3, 4].

Tumors with the greatest prevalence of *CDKN2A* loss include malignant gliomas [5–7], lung adenocarcinoma, pancreatic adenocarcinoma, melanoma and bladder urothelial carcinoma [8]. Among brain tumors, *CDKN2A* loss has greatest clinical implications in histologically low and intermediate grade gliomas and meningiomas [1]. In IDH-mutant astrocytomas, the presence of homozygous deletion of *CDKN2A* is associated with poor outcome and an expected median overall survival of only 3 years [9–12]. Hence, *CDKN2A* homozygous deletion is now considered a CNS WHO grade 4 diagnostic marker in IDH-mutant astrocytomas, even in the absence of necrosis and/or microvascular proliferation on histology [1]. While 1p/19q co-deletion and IDH mutation occur in the early stages of oligodendroglioma formation, progression to a higher grade is associated with homozygous deletion of *CDKN2A*/*B*. Less than 10% of CNS WHO grade 3 IDH-mutant and 1p/19-codeleted oligodendrogliomas show homozygous *CDKN2A/B* deletion, associated with worse outcome and shorter overall survival [9]. Since *CDKN2A* loss is not seen in low-grade IDH-mutant oligodendrogliomas, detection of this molecular alteration can be used to distinguish between grade 2 and grade 3 tumors when histology is equivocal. In pediatric low-grade gliomas, the frequency of *CDKN2A/B* loss varies from 6 to 20% [13, 14]. It is more prevalent in BRAFV600E mutant tumors. The co-existence of mutant BRAFV600E with *CDKN2A* loss suggests transformation into histologically higher-grade brain tumor, with more aggressive behavior and worse clinical course [13, 15, 16]. In pilocytic astrocytomas, *CDKN2A* inactivation also heralds more aggressive clinical behavior [13]; its presence in astrocytomas with piloid features is now suggestive of a distinct high-grade glioma subtype [1, 17]. Similarly, in meningiomas *CDKN2A* homozygous loss is associated with anaplastic histology and with increased risk of recurrence or progression [18–22]. It is now considered a diagnostic marker of grade 3 in meningioma, independent of histology [1].

While the presence of *CDKN2A* loss is gaining increasing recognition as a key diagnostic and prognostic marker in gliomas and meningiomas, and is an inclusion criterion for some clinical trials, its molecular detection remains expensive, time consuming and not widely available. Testing for loss of expression in p16, the protein product of *CDKN2A*, by immunohistochemistry provides a simpler and low-cost alternative to *CDKN2A* molecular testing. There are limited studies correlating p16 protein expression by immunohistochemistry with the presence of *CDKN2A* loss [23–28], primarily using PCR or FISH-based determination of *CDKN2A* status. None so far have established a cutoff value for the sensitivity or specificity of p16 as a surrogate marker for homozygous loss of *CDKN2A* detected using highly sensitive next-generation DNA sequencing [29]. This study performs semi-quantitative analysis for p16 expression across 100 IDH-wildtype and IDH-mutant gliomas, using three independent p16 immunoreactivity scores, and correlates the extent of p16 expression with *CDKN2A* homozygous deletion as determined by next-generation DNA sequencing. It establishes p16 as a reliable, highly sensitive surrogate marker for inference of *CDKN2A* homozygous deletion in gliomas, with a recommended p16 expression score of ≤ 5% for confirming and > 20% for excluding *CDKN2A* homozygous loss.

## Materials and methods

### Samples

A cohort of 100 glioma cases diagnosed from 2019 to 2022 at the Mount Sinai Health System (MSHS) were selected for the study, and were used under approved institutional review board protocol. The combined histopathologic / molecular integrated diagnosis was based on guidelines from the 5th edition (2021) of the WHO classification of CNS tumors [1].

### *CDKN2A* status

The *CDKN2A* status for all cases was determined by a reference laboratory, FoundationOne®CDx (F1CDx), utilizing highly sensitive hybrid capture-based next-generation DNA sequencing technology and a customized pipeline to detect genomic alterations, including copy number alterations (CNA) such as amplifications and homozygous deletions [29, 30]. Briefly, to detect CNAs, a log-ratio profile of the sample was obtained by normalizing the overall sequence coverage against a process-matched normal control. This profile was then corrected for GC bias, segmented, and used to estimate copy number at each segment, purity- and ploidy-adjusted [29, 30]. The threshold for calling homozygous deletions was copy number in the tumor equal to zero. All cases had tumor content of 20% or greater.

### P16 scoring

P16 immunohistochemical analysis was performed on 4 µm-thick formalin fixed and paraffin embedded sections, using the most widely used E6H4 clone of the anti-p16 mouse monoclonal primary antibody (Roche CINtec Histology, 725–4793) on the automated Ventana Ultra immunohistochemical staining system with the following optimized conditions: heat-induced epitope antigen retrieval for 172 min using CC1 buffer at high pH, pre-diluted primary antibody incubation for 32 min, and the UltraView DAB detection kit (Roche, 760–500). Whole digital slide images, from different tumor areas in most cases, were available for evaluation. Initially, two pathologists scored the percentage of p16-positive tumor cells in a blinded study with no knowledge of the histological diagnosis or molecular CDKN2A status. This was followed by a second, unblinded consensus evaluation for selected “gray-zone” cases with 6–20% p16 expression. Nuclear and/or cytoplasmic staining of tumor cells was considered as positive staining. P16 expression within each tumor was calculated based on the average of a maximum and a minimum tumor percentage score, obtained at 10X microscopic fields with highest tumor cellularity. Additionally, QuPath [31] bioimage analysis was used as an unbiased digital quantification method of p16 expression, performed on 99 of the 100 tumors, using the same 10X fields scored by pathologists. The QuPath setup for image detection was established for brightfield with positive detection by optical density, using the following settings: 0.05 background intensity parameter, 0.1 single threshold, using compartment score at a mean optical density of nuclear DAB staining.

### Statistics

PRISM was used for graph and receiver operating characteristic (ROC) curve generation and for all statistical calculations. Statistical significance for p16 expression in non vs. *CDKN2A* homozygous deleted tumors was determined using one-way ANOVA with Brown-Forsythe test correction, as well as using unpaired two-tailed Student t-test. The area under the ROC curve (AUC or C-index) was calculated to measure correlation between p16 score and *CDKN2A* status, with a perfect correlation considered as area = 1.0 and a random one considered as area = 0.5. A two-tailed p-value was computed using a z ratio of (AUC – 0.5) over the standard error. Statistical significance was considered at a level of *p* < 0.05.

## Results

We quantified p16 expression by immunohistochemistry in 100 gliomas with diverse histological features and grades, for which *CDKN2A* status was determined using highly sensitive, targeted DNA-based hybridization capture next-generation sequencing technology. The histologic diagnoses included: Astrocytoma, IDH-mutant, WHO grade 2–4; Oligodendroglioma, IDH-mutant and 1p/19q-codeleted, WHO grade 2–3; Glioblastoma, IDH-wildtype, WHO grade 4; Pilocytic astrocytoma, WHO grade 1; Low-grade glioneuronal tumor/pleomorphic xanthoastrocytoma; Angiocentric glioma, WHO grade 1; Diffuse hemispheric glioma, H3G34-mutant, WHO grade 4; Diffuse low-grade glioma, MAPK pathway-altered; and Diffuse pediatric-type high-grade glioma, H3-wildtype and IDH-wildtype (Table 1, Additional file 1: Data 1). The cohort ages ranged from 2 to 85 years, with a median age of 54 years and only a slight male preponderance of 51% (Table 1). The majority of cases were primary resections (79%).

**Table 1:** Clinical features and histological type of glioma cohort (n = 100)

Age (years)	2–85
Sex	
Male	51
Female	49
Surgery	
Biopsy	11
Resection	89
Presentation	
Primary	79
Recurrence	21
IDH status	
IDH-mutant	29
IDH-wildtype	71
Histologic subtype	
Astrocytoma, IDH-mutant, CNS WHO grade 2	3
Astrocytoma, IDH-mutant, CNS WHO grade 3	12
Astrocytoma, IDH-mutant, CNS WHO grade 4	5
Oligodendroglioma, IDH-mutant and 1p/19q-codeleted, CNS WHO grade 2	5
Oligodendroglioma, IDH-mutant and 1p/19q-codeleted, CNS WHO grade 3	3
Glioblastoma, IDH-wildtype, CNS WHO grade 4	62
Pilocytic astrocytoma, CNS WHO grade 1	5
Low-grade glioneuronal tumor / Pleomorphic Xanthoastrocytoma	1
Angiocentric glioma, CNS WHO grade 1	1
Diffuse hemispheric glioma, H3 G34-mutant, CNS WHO grade 4	1
Diffuse low-grade glioma, MAPK pathway-altered	1
Diffuse pediatric-type high-grade glioma, H3-wildtype and IDH-wildtype	1

**Fig. 1:**
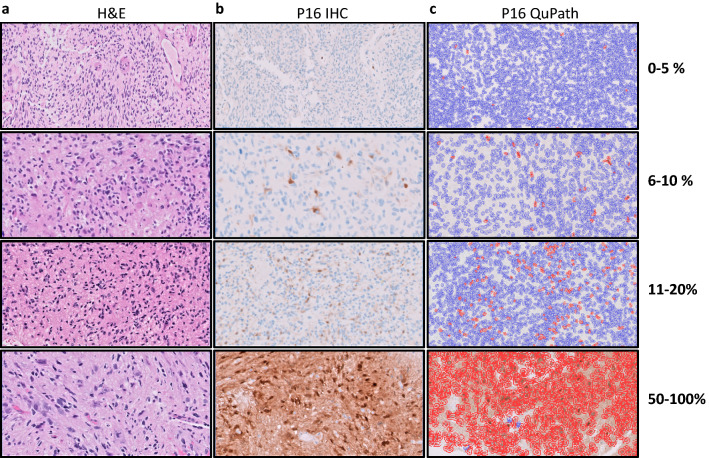
P16 expression analysis in gliomas of representative score ranges **a** Hematoxylin and eosin (H&E)-stained gliomas **b** P16 immunohistochemistry (IHC) used for semi-quantitative analysis of p16 expression, in tumors from part A (brown represents positively stained cells). **c** Digital quantification of p16 immunohistochemistry using QuPath (red represents positively stained cells, blue represents negative cells). 20X magnification (top row; 0–5%), 40X magnification (remaining images, 6–100%)

**Fig. 2:**
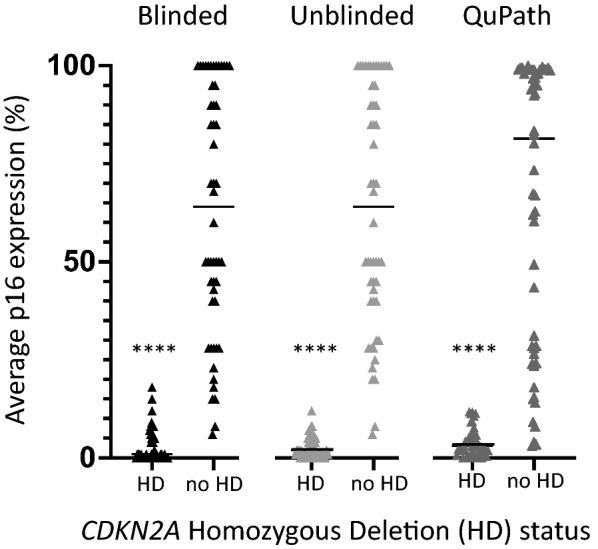
Analysis of p16 expression in glioma cohort *CDKN2A* status was determined by next-generation sequencing in 100 brain tumors. P16 expression was quantified using blinded pathologist, unblinded consensus pathologist, and QuPath digital pathology. Line represents median, *p*-values calculated by one-way ANOVA and unpaired Student t-test (****p < 0.0001 using all tests)

**Fig. 3:**
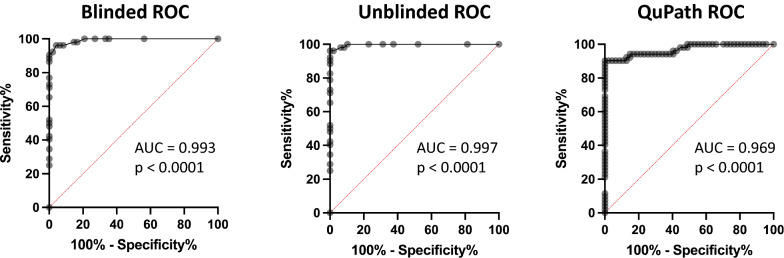
Receiver operating characteristic curve analysis for test performance in Blinded, Unblinded consensus, and QuPath p16 scoring methods. Area under the curve (AUC) and *p*-value are provided for each, calculated using Wilson/Brown test

P16 expression was determined as the average of the minimum and maximum percent tumor cell staining, counted in a 10X microscopic field of an area with highest tumor cellularity. This was done manually in both blinded and unblinded consensus reviews by two pathologists (Figs. 1a, b, 2, Additional file 1: Data 1) and digitally via QuPath analysis (Figs. 1c, 2, Additional file 1: Data 1). Immunoreactivity for p16 in non-neoplastic endothelial cells and/or neurons, when recognized with high confidence by the pathologists, was excluded from the final tumor score in the blinded and unblinded pathologist analyses. Overall, all p16 quantification methods showed concordant results (Fig. 2). In all three, there was significant difference (*p* < 0.0001, one-way ANOVA and *t*-test) in p16 expression between tumors with *CDKN2A* homozygous deletion (HD) and those without (Fig. 2). Classifying *CDKN2A* status based on p16 tumor cell expression (0–100%) demonstrated robust performance over a wide range of thresholds, with receiver operating characteristic (ROC) area under the curve (AUC) of 0.993 and 0.997 (blinded and unblinded consensus pathologist p16 scores, respectively) and 0.969 (QuPath p16 score) (Fig. 3).

**Fig. 4:**
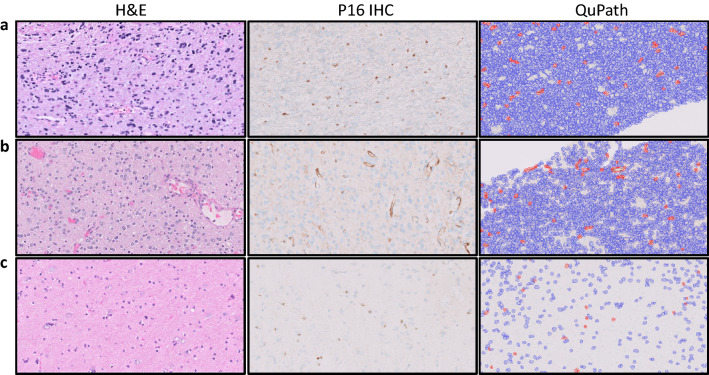
Case examples with imperfect correlation between p16 expression and *CDKN2A* homozygous loss. **a** H&E, P16 IHC, and QuPath analyses in a tumor with *CDKN2A* homozygous loss, overscored due to retained p16 staining in neurons. **b** H&E, P16 IHC, and QuPath analyses in a tumor with *CDKN2A* homozygous loss, and retained p16 staining in endothelium. **c** H&E, P16 IHC, and QuPath analyses in a tumor without *CDKN2A* homozygous loss, underscored due to low tumor cellularity. H&E = Hematoxylin and eosin; IHC = Immunohistochemistry. 40X magnification (**a**–**c**)

Expression of p16 as scored by pathologists was also correlated with *CDKN2A* status by grouping tumors into one of five categories based on a range of p16 percent expression: 0–5%, 6–10%, 11–20%, 21–50% and 51–100% (Table 2). Notably, all tumors with 0–5% p16 expression carried *CDKN2A* HD and were thus considered as true positives. Similarly, all tumors with 21–100% p16 expression did not carry *CDKN2A* HD and were considered as true negative. In contrast, tumors falling within the 6–10% and 11–20% range (gray zone) showed an imperfect correlation to *CDKN2A* status. To evaluate tumors in this range further, we unblinded our results and re-scored tumors based on consensus discussion and additional criteria (re-evaluation of tumor cellularity on H&E with exclusion of mostly normal-appearing areas, additional p16 staining where suboptimal, consideration of weak cytoplasmic staining as positive, recognition and exclusion of background non-neoplastic staining) (Figs. 2 and 4). Unblinded consensus rescoring resulted in a slight decrease of gray zone cases: the number of tumors in the 6–10% range went from 9 to 6, with only two false positive results; and the tumors in the 11–20% range went from 7 to 3, with only one false negative result (Table 2). Examples of cases initially overscored in the blinded analysis included a tumor with *CDKN2A* HD, which showed retained p16 staining in scattered entrapped non-neoplastic cells (Fig. 4a and b). Few tumors without *CDKN2A* HD remained underscored even after unblinded consensus analysis, due to low tumor cellularity (Fig. 4c). Consensus re-scoring did not alter the overall trend for tumors within the 0–5% and 21–100% ranges.

**Table 2:** *CDKN2A* homozygous deletion status in tumors grouped by pathologist-scored p16 score ranges

P16 score	Blinded p16 analysis		Unblinded consensus p16 analysis	
	*CDKN2A* HD	No *CDKN2A HD*	*CDKN2A* HD	No *CDKN2A HD*
0–5%	38	0	43	0
**6–10%**	7	2	4	2
**11–20%**	3	4	1	2
21–50%	0	19	0	21
51–100%	0	27	0	27

*HD* Homozygous deletion. P16 score  bold values indicate gray zone ranges containing false positives and negatives

Next, diagnostic test metrics were assessed by defining false negatives and positives as determined in Table 2, calculated at different p16 cutoffs. In the blinded pathologist-based p16 scoring with a p16 cutoff value of 10%, overall test sensitivity was 94% and test specificity was 96%, with a positive predictive value (PPV) of 96% and a negative predictive value (NPV) of 94%. With a cutoff value of 5%, overall test sensitivity decreased to 79% and NPV decreased to 84%, while specificity and PPV increased to 100%. Unblinded consensus-score analysis improved the blinded-score analysis test sensitivity to 98% and 90%, at p16 score cutoff values of 10% and 5%, respectively. QuPath-based p16 scoring showed overall similar trends for increased test specificity and PPV with decreasing cutoffs (94% specificity and 92% PPV at 5% cutoff vs. 90% specificity and 89% PPV at 10% cutoff), at the expense of test sensitivity (89% at 10% cutoff vs. 77% at 5% cutoff) (Additional file 1: Data 1). Taking into account pathologists’ blinded and unblinded consensus p16 scored case distribution and a more conservative threshold that optimizes test specificity and PPV, we conclude the likelihood of a homozygous *CDKN2A* deletion to be very likely when p16 expression is 0–5%, and similarly, very unlikely when p16 expression is above 20%. In contrast, 6–20% range of p16 expression in gliomas represents a gray zone where molecular testing is still helpful to confirm true *CDKN2A* status.

## Discussion

While *CDKN2A* homozygous deletion (HD) has been recognized as both a diagnostic and a prognostic marker in gliomas and meningiomas, its detection is not widely accessible and cost effective. In this current study, we examined whether simple quantification of p16 immunoreactivity can serve as a surrogate marker for *CDKN2A* loss in gliomas. Our results demonstrate strong correlation between the degree of p16 immunostaining and the presence of *CDKN2A* HD across IDH-wildtype and IDH-mutant tumors of all grades. In tumors with pathologist-scored p16 greater than 20%, we found 100% specificity for excluding *CDKN2A* HD, and in tumors with p16 equal to or less than 5%, we found 100% specificity for predicting *CDKN2A* HD. Thereby, our study provides a cost effective and convenient method for evaluating *CDKN2A* homozygous loss status in glioma, as an alternative to expensive genomic sequencing.

Our results build on several prior studies, which use FISH or PCR to detect *CDKN2A* gene copy loss and immunohistochemistry to correlate with p16 expression, many of them using the same antibody clone [23–28]. Earliest studies by Rao et al. and Burns et al. used multiplex PCR to detect *CDKN2A* deletion in brain tumors and correlated it with p16 expression in astrocytomas, where a strong correlation was found between p16 negative tumors and homozygous loss of *CDKN2A* [23]; as well as in glioblastomas, where diffuse p16 immunostaining was found to confidently exclude *CDKN2A* deletion but p16 immunonegativity did not always correlate with *CDKN2A* deletion [24]. A following study by Parkait et al. did find significant association between p16 immunonegativity and *CDKN2A* deletion detected by FISH in glioblastoma [25]. Subsequently, Park et al. found only moderate correlation between p16 expression (performed on tissue microarrays) and *CDKN2A* loss as determined by FISH, but demonstrated the strong prognostic value of p16 expression in IDH-mutant astrocytomas [26]. Most recently, Suman et al. and Geyer et al. showed evidence for the strong negative predictive value of p16 in detecting *CDKN2A* deletion, also using FISH for determining *CDKN2A* status [27, 28]. Some of the reported limitations in the above studies include false positive FISH results due to partial hybridization failure, artifacts, and sub-optimal p16 cutoff values, hampering the standardized use of p16 as a surrogate marker for *CDKN2A* homozygous deletion in gliomas.

By leveraging the superior sensitivity of next-generation DNA sequencing [29] with semi-quantitative scoring methodologies and digital pathology, our study puts forward specific threshold values for p16 expression as a surrogate marker of *CDKN2A* HD status, enabling greater standardization of this cost-effective tool in glioma diagnostics. Given the diagnostic and prognostic implications when *CDKN2A* HD is detected in a lower grade glioma, we favored a conservative threshold p16 expression value of 5%, which optimizes both test specificity and positive predictive value for *CDKN2A* HD detection, over a threshold of 10% or higher, which leads to occasional overcalling of *CDKN2A* HD (i.e. false positives). By introducing a second cutoff of 20% for the exclusion of homozygous loss and continuing to sequence cases within the 6–20% gray zone, we find virtually perfect concordance between pathologist-scored p16 expression and *CDKN2A* HD status, without any false positives or false negatives.

Recently, an analogous analysis in meningiomas by Tang et al. showed that loss of p16 expression is a sensitive marker of *CDKN2A* loss determined by next-generation sequencing [32]. Similarly to meningiomas, *CDKN2A* HD is a molecular signature for highest grade in IDH-mutant astrocytomas (grade 4) and in IDH-mutant and 1p/19q-codeleted oligodendroglioma (grade 3), regardless of histology [1]. In our cohort, 3 out of 20 IDH-mutant astrocytomas and none out of 8 IDH-mutant oligodendrogliomas contained *CDKN2A* HD, overall consistent with prior reported frequencies [9] (Additional file 1: Data 2). Importantly, the presence of *CDKN2A* HD (with pathologists’ p16 score of 1%) upgraded one IDH-mutant astrocytoma without microvascular proliferation or palisading necrosis to grade 4 (Additional file 1: Data 1). Moreover, *CDKN2A* HD was detected in 1 out of 5 pilocytic astrocytomas (with pathologists’ p16 score of 1–2%). This pilocytic astrocytoma displayed atypical features, including elevated mitotic activity and increased MIB1 proliferation index, as well as an aggressive clinical behavior with recurrence only 10 months after initial resection. Of note, the tumor classified as a posterior fossa pilocytic astrocytoma rather than a high-grade astrocytoma with piloid features by orthogonal DNA methylation analysis. This confirms the diagnostic and prognostic value of *CDKN2A* HD as previously established [1, 13]. As p16 in both cases was less than 5%, it further demonstrates the utility of p16 as a surrogate marker of *CDKN2A* HD in clinical neuropathology, enabling quicker final diagnosis and circumventing expensive molecular testing.

Our study is not without limitations. While we found perfect correlation between *CDKN2A* HD status and pathologist-scored p16 expression in the 0–5% and 21–100% p16 score ranges, sensitivity and specificity were lower in the 6–20% range (so-called gray zone) with several false positive and false negative cases present in this range. A few of the cases in this gray zone were moved to the 0–5% and 21–100% ranges after unblinded consensus re-scoring. For example, two *CDKN2A* HD cases in the blinded study were over scored, but consensus discussion deemed the positive p16 staining to be mostly limited to neurons and/or glia (Fig. 4a) or endothelial cells (Fig. 4b). These examples highlight the potential confounding factor of background non-neoplastic brain tissue, which has been previously reported to show nuclear and cytoplasmic reactivity for p16 in scattered astrocytes, OPCs, and/or neurons, related to cellular senescence [33–35]. In our own experience with p16, we have observed occasional and inconsistent immunoreactivity in only scattered glia, neurons, and endothelium. To minimize non-neoplastic background in our scores, we evaluated the most densely cellular tumor area, correlated it to its H&E, and subtracted p16 reactivity when confidently recognized as endothelial or neuronal. We cannot exclude the possibility of rare p16 reactivity contributed by entrapped non-neoplastic glia within the tumor bulk, as reactive and neoplastic glia are extremely challenging to discriminate. A pattern of p16 staining in which positive cells are scarce and equally distributed from one another, rather than overlapping and clustering, was suggestive of non-neoplastic background (Fig. 4a). Importantly, QuPath analysis was unable to perform a similar background subtraction. Conversely, few cases without *CDKN2A* HD were found to be under scored after unblinding our analyses. This was most often due to the tumor representing a small biopsy composed of mostly normal brain with only few tumor cells at the infiltrative edge in an otherwise low-grade glioma (Fig. 4c). Even after unblinding ourselves to *CDKN2A* status, such cases remained in the gray zone, as we could not confidently distinguish normal from neoplastic cells. QuPath analysis also underscored p16 expression in such tumors (Fig. 4c). Thus, areas of high tumor cellularity may be necessary for interpretation of p16 immunoreactivity, as it is hard to discriminate scattered infiltrating tumor cells amidst mostly non-neoplastic glia, especially in small biopsy specimens and when using digital software for scoring.

Another caveat in correlating p16 expression to *CDKN2A* inactivation are the occasional tumors in which p16 expression is lost due to epigenetic silencing of the *CDKN2A* locus, rather than homozygous deletion [2]. We cannot exclude that some of the false positive cases in the 6–20% gray zone may indeed have had inactivated *CDKN2A* transcription through an epigenetic mechanism, leading to the loss of p16 expression in the absence of genomic loss at the 9p21 locus. This caveat is especially important to consider in tumors with global epigenetic alterations. Thus, our study concludes a strong correlation between p16 expression and *CDKN2A* homozygous deletion, rather than between p16 expression and *CDKN2A* inactivation. Finally, while the utilized next-generation sequencing technology has high sensitivity for capturing homozygous *CDKN2A* loss with lower false positives compared to FISH, it did not include calls for tumors with a single allele (hemizygous) *CDKN2A* loss. Indeed, we cannot exclude that some of the cases without *CDKN2A* homozygous deletion may have had loss of one of the *CDKN2A* alleles. Given that *CDKN2A* encodes tumor suppressors and the current literature correlates only homozygous *CDKN2A* loss with prognosis and grade in gliomas and meningiomas, determining hemizygous loss in our cohort was deemed irrelevant. In all, this study supports other recent findings [23–28, 32] for the role of p16 as a surrogate marker of *CDKN2A* loss, and establishes a cutoff p16 value of 5% for detecting homozygous *CDKN2A* deletion with robust sensitivity and specificity, and a cutoff p16 value of 20% for excluding homozygous *CDKN2A* deletion, in both low and high-grade gliomas.

## Supplementary Information

**Additional file 1: Data 1**. Metadata file including de-identified patients’ demographics, tumor characteristics, average p16 scores, and molecular CDKN2A status. **Data 2**. Frequency of CDKN2A homozygous deletion in tumors of different histology and grade.

## Data Availability

The data that support the findings in this study are provided as Additional file 1: Data 1.
